# Assessing the treatment pattern, health care resource utilisation, and economic burden of multiple myeloma in France using the Système National des Données de Santé (SNDS) database: a retrospective cohort study

**DOI:** 10.1007/s10198-022-01463-9

**Published:** 2022-05-25

**Authors:** Antoine Bessou, Xavier Colin, Julie De Nascimento, Will Sopwith, Shannon Ferrante, Boris Gorsh, Benjamin Gutierrez, Leah Sansbury, Jenny Willson, Sandhya Sapra, Prani Paka, Feng Wang

**Affiliations:** 1 IQVIA, Paris, France; 2 GlaxoSmithKline, Rueil-Malmaison, France; 3 IQVIA, London, UK; 4 Value Evidence and Outcomes, GlaxoSmithKline, Upper Providence, Collegeville, PA USA; 5 Value Evidence and Outcomes, GlaxoSmithKline, Research Triangle Park, Durham, NC USA; 6 Value Evidence and Outcomes, GlaxoSmithKline, London, UK; 7 Global Medical Affairs, GlaxoSmithKline, Upper Providence, Collegeville, PA USA

**Keywords:** Multiple myeloma, HCRU, Cost, Line of therapy, Economic burden, France

## Abstract

**Background:**

Real-world data on health care resource utilisation (HCRU) and costs for French patients with multiple myeloma (MM) are limited due to the quickly evolving MM treatment landscape. This retrospective, national-level study quantified the MM economic burden in France.

**Methods:**

The study included patients with newly diagnosed MM from the Système National des Données de Santé coverage claims database between 2013 and 2018 who received active treatment within 30 days of diagnosis. HCRU included hospitalisations, drugs, consultations, procedures, tests, devices, transport, and sick leave. Costs were annualized to 2019 prices. Drug treatments, reported by line of therapy (LOT), were algorithmically defined using drug regimen, duration of therapy, and gaps between treatments. Analyses were stratified by stem cell transplantation status and LOT.

**Results:**

Among 6413 eligible patients, 6229 (97.1%) received ≥ 1 identifiable LOT; most received 1 (39.8%) or 2 LOT (27.5%) during follow-up. Average annual hospitalisation was 6.3 episodes/patient/year (median duration: 11.6 days). The average annual cost/patient was €58.3 K. Key cost drivers were treatment (€28.2 K; 39.5% of total HCRU within one year of MM diagnosis) and hospitalisations (€22.2 K; 48.6% of total HCRU costs in first year). Monthly treatment-related costs increased from LOT1 (€2.447 K) and LOT5 + (€7.026 K); only 9% of patients received LOT5 + . At LOT4 + , 37 distinct regimens were identified. Hospitalisation costs were higher in patients with stem cell transplantation than total population, particularly in the first year.

**Conclusions:**

This study showed a high economic burden of MM in France (€72.37 K/patient/year in the first year) and the diversity of regimens used in late-line treatments.

**Supplementary Information:**

The online version contains supplementary material available at 10.1007/s10198-022-01463-9.

## Introduction

The incidence of multiple myeloma (MM) is increasing globally [1]. Western Europe is one of 3 regions with the highest age-standardised incidence of MM globally [1]. According to estimates, in 2020 France had the second-highest MM burden in the European Union (EU), with 10 cases per 100,000 population, compared with an average of 7.5 cases per 100,000 in the EU [2].

The MM treatment landscape is changing rapidly, and major advances have been made in the treatment of patients with MM in the last decade, including an increase in the number of approved novel drugs and use of combination treatments [3]. Therapies including monoclonal antibodies and advanced generations of proteasome inhibitors and immunomodulatory agents have significantly improved patient outcomes, including response rates and duration of progression-free and overall survival, when compared with conventional treatments [4]. For most patients, however, MM remains incurable and patients often repeatedly relapse, with a worsening prognosis and shorter duration of treatment response with each subsequent relapse [5–8]. The distribution of patients newly diagnosed with MM ISS stage 1 (20–24%), stage 2 (38–44%), or stage 3 (33–39%) disease reflect the severity of MM at diagnosis [9, 10]. The median duration from diagnosis to first relapse is around 22.7 months [11]. The median overall survival for patients receiving first, second, third, and fourth line of treatment (LOT) was reported to be 37.5, 19.7, 13.9, and 9.2 months, respectively [12].

Patients often require multiple LOTs, which come with increased costs. Currently, there is no standard of care treatment for relapsed/refractory MM (RRMM). With the emergence of more novel treatments and combination therapies, treatment decisions are likely to become even more complex. Although these new treatments offer improved care, they may be associated with higher health care costs.

Drivers of the costs of managing patients with MM include stem cell transplantation (SCT), multiple drug regimens over the course of the disease, tests, and repeated hospitalisations. Current real-world data on health care resource utilisation (HCRU) costs for patients with MM in France, and in Europe in general, are limited, particularly for patients with heavily pretreated RRMM. In one estimate, the reimbursed costs of care for patients with MM or malignant tumour plasma cells reached ~ 1 billion Euros (€) in France [13]. Studies conducted in Europe between 2001 and 2015 have shown drug and hospitalisation costs to be the largest components of total HCRU-associated costs and that costs vary by LOT [14–18]. However, because the most recently approved drugs were not included, these analyses may not reflect current HCRU and costs.

Understanding patient HCRU patterns, disease burden, and health-related expenditure is important when evaluating the potential value of new treatments and facilitates targeted improvements in MM management. Analyses of health insurance databases can guide public health care decisions, monitor various types of medical expenditures, inform epidemiological studies, evaluate medical practices or health system experimentations, and can be used for international comparisons [19]. The aim of this study is to describe the treatment patterns, quantify the MM economic burden in France, and identify HCRU associated with MM treatment using the Système National des Données de Santé (SNDS) national coverage claims database.

## Methods

### Study overview and data source

This descriptive, retrospective cohort study used claims data in the SNDS across all regions of France, except from those affiliated with an institution in Mayotte. The database includes reimbursement claims data covering at least 99% of French residents [20]. Information is held on outpatient claims, hospital discharges, deaths, and disabilities, with records linked by pseudonymised record identification [19, 21]. Diagnoses are coded according to the International Classification of Diseases version 10 (ICD-10) and medical procedures according to the Classification Commune des Actes Médicaux [CCAM]).

The study sponsors (i.e., the authors affiliated with GSK) initiated the study by contracting IQVIA France to access the SNDS database, collect and perform analysis on raw data, and develop a study report. In accordance with French medical data privacy laws, IQVIA acquired access to the SNDS database, which required ethics approval, and approval from the Comité d'Expertise pour les Recherches, les Etudes et les Evaluations dans le domaine de la Santé (CESREES) and the Commission Nationale de l'Informatique et des Libertés (CNIL). Data access was delivered by the Caisse nationale d'assurance maladie (CNAM) after signing an agreement.

### Study population

**Fig. 1:**
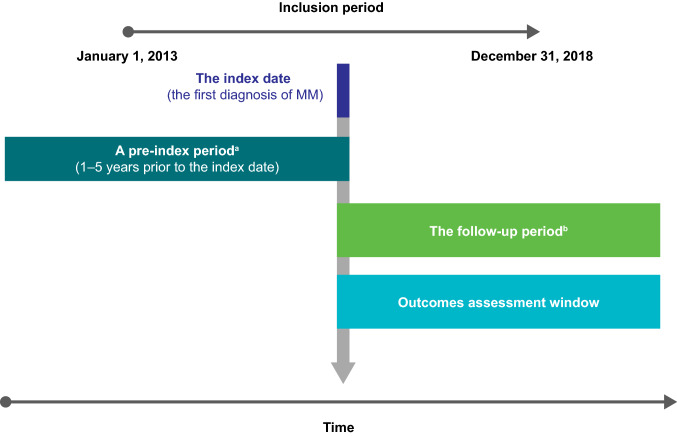
Schema of study parameters for inclusion of newly diagnosed patients with MM and outcomes assessment. ^a^Used for the description of patients at baseline, comorbidities, or confirming incident cases; ^b^The time between index date and death, end of follow-up (a gap of 12 months without reimbursed care in SNDS), or the end of the study inclusion period. *MM* multiple myeloma, *SNDS* Système National des Données de Santé

Eligible patients from the SNDS database had a new diagnosis of MM between January 1, 2013, and December 31, 2018, were older than 18 years, and were undergoing active treatment for MM (i.e., treated within 30 days after initial diagnosis) (Fig. 1). To minimize the potential erroneous inclusion of patients with coding errors and align with other SNDS study designs, diagnosis of MM was identified by at least 2 records of MM diagnosis: ICD-10 codes from a primary, related or associated diagnosis (C90, C90.x) during hospital stays, or a MM diagnosis as long-term disease during the study time period (or both). Historical data for these patients were available from January 1, 2008. To maximise patient inclusion, minimum time of follow-up was not specified. In addition, patients were required to have a minimum of 1 year of history in the SNDS prior to MM diagnosis and be affiliated with the general reimbursement scheme, which captures salaried employees in the private sector and their dependents, representing about 76% of French inhabitants in 2015 [19]. Patients with a diagnosis of MM within 1 year before the index date (defined as the first diagnosis of MM recorded during the inclusion period) or with another malignancy (except for non-myeloma skin cancer) within 5 years of the index date were not eligible.

### Inputs

All inputs, including HCRU and cost, were extracted directly from the SNDS database. Inputs included patient demographics and clinical characteristics, as well as exposures including LOT, SCT, and adverse events (AEs). Demographic and clinical characteristics were defined using the most recent record prior to the index date. Dispensed medications were identified using Anatomical Therapeutic Chemical codes, and outputs included date of prescribing, date of dispensing, setting of prescribing, prescriber specialty, and number of packs dispensed.

LOT was algorithmically defined based on published criteria using information on the drug regimen, time since first administration, and a gap between treatment regimens (Supplementary Fig. 1) [22, 23]. Drugs had to be approved for MM before December 2018. All drugs dispensed within 28 days following the treatment initiation date were considered first-line therapy (LOT1). A LOT was defined as continuing until a new drug was added (excluding widely used therapies, such as corticosteroids) or the discontinuation of all drugs in the LOT, defined as a treatment gap of at least 90 days following the end of the grace period. A grace period was allowed between 2 successive administrations of drug, based on the usual duration of a full prescription administered in clinical practice in France and adapted from Palmaro et al. 2017 [23]. Date of discontinuation in this case was defined as the date the grace period ended.

Fully observable drugs in the SNDS were defined as “high-cost drugs,” costly and innovative drugs dispensed in the hospital which are excluded from the diagnosis-related group system; “temporarily authorised drugs,” exceptional hospital use of products without marketing authorisation; “retrocession drugs,” dispensed to ambulatory patients within the hospital; or “drugs in community,” only available in community settings (Supplementary Table 1). Some drugs (e.g., melphalan and cyclophosphamide) that can be administered intravenously or orally were partially observable in SNDS; community-based oral administration was observable, but hospital-based intravenous administration was not.

Treatment regimens incorporating drugs that were not observable in the SNDS, e.g., corticosteroids, were constructed from assumptions based on treatment recommendation guidelines [23, 24]. SCT was determined by hospitalisation with a related diagnosis-related group code or a procedure CCAM code (Supplementary Table 2). Relevant AEs for all newly diagnosed patients with MM were identified using ICD-10 codes from hospital diagnoses (Supplementary Table 3).

### Outcomes

All-cause HCRU was assessed for the following categories: private and public hospitalisations, outpatient physician and paramedic visits, medical procedures, laboratory tests, dispensing of observable drugs and medical devices, financial sickness benefits, invalidity pensions, and reimbursed transport expenses.

Events of interest (AEs and comorbidities) included keratopathy/keratitis, blurred vision, cataracts, glaucoma, light sensitivity, bleeding events, infusion reaction, dry eye, anaemia, neutropenia, thrombocytopenia, infection, blood clots, skeletal-related events, peripheral neuropathy, venous thromboembolism, diarrhoea, shingles, and pneumonia. HCRU related to events of interest was assessed for patients with incident MM from index date to end of follow-up and included hospitalisations with any of these conditions when associated with a primary diagnosis. Each hospitalisation based on a single primary diagnosis of an event of interest was counted as a unique event. The results were reported as the proportion of the study population experiencing each event and the rate per patient per year (PPPY). To adjust for varying lengths of follow-up time, costs and healthcare use were also reported as a mean Per Patient Per Month (PPPM). PPPM costs were calculated by summing all costs incurred during the observation period divided by the sum of length of the observation period for each patient.

Costs of HCRU related to the administration of MM treatment were classified as MM-related treatment administration. These included costs incurred within hospital, such as costs of “high-cost drugs” indicated for MM, all stays for which the main diagnosis indicated a chemotherapy session and a related diagnosis of MM (entire cost of stay was counted), and transport following MM-related treatment or hospital stay. Costs incurred as an outpatient, including cost of drugs with an indication for MM, and sick leave within 7 days following MM-related treatment or hospital stay were also classified as MM-related treatment administration costs.

HCRU and costs related directly to MM included those described for MM-related treatment administration plus all stays with a primary or associated diagnosis of MM, all rehabilitation stays directly following a MM related stay, and all home hospitalisation with a diagnosis of MM. Outpatient laboratory tests, imaging, physician visits, and medical procedures and devices were excluded from this analysis.

### Data analyses

Costs of HCRU for each category were annualised to 2019 prices and quoted in € PPPY or per patient per month (PPPM). Cost rate was based on the total cost of a specific HCRU during the follow-up period divided by the number of person-years available for analysis, regardless of individual HCRU exposure. Descriptive analyses were performed using number and percent for categorical variables, and mean, standard deviation (SD), median, interquartile range (IQR), as well as minimum and maximum for continuous variables.

Analyses were stratified by SCT status and LOT. Treatments were analysed and reported as number and percentage of patients receiving each treatment drug and regimen by LOT, with later lines (LOT5 and above) aggregated. Duration of each LOT was calculated both descriptively (from first treatment administered until end of LOT) and using Kaplan–Meier analysis. Patients were censored at the end of follow-up period, loss to follow-up, or death. A Charlson comorbidity index was calculated based on a published study, using patients’ comorbidities recorded at the index date and during the year preceding the index date [25].

## Results

### Patient characteristics

Among 58,903 patients with MM diagnosis identified in the SNDS database during the inclusion period, 44,421 had either at least 2 records of MM diagnosis during hospital stays or at least 1 record during a hospital stay and 1 record of long-term disease. Of these, 25,717 patients had incident MM and 6413 patients met study eligibility criteria (Supplementary Fig. 2). Of the study eligible patients, 6257 (97.6%) had ≥ 5 years of data history prior to the index date. A total of 15,751 (26.7%) patients were excluded based on drug treatment status; 6914 (11.7%) received no treatment during follow-up and a further 8837 (15%) received no treatment within 30 days after diagnosis. The mean age at index date was 68.9 years (SD, 11.67), and 52.1% were male (Table 1). Patients were distributed across all administrative regions of France. Patient enrolment was also distributed across index years (Supplementary Table 4). Median follow-up was 22 months (IQR, 29). Two-thirds of patients were still alive at the end of the study period (*n* = 4217; 65.8%); 2167 patients (33.8%) died during follow-up, and 29 patients (0.5%) were lost to follow-up or disenrolled (made no claims in the period of 1 year).

**Table 1:** Baseline characteristics and patient disposition

	Patients with SCT (*n* = 1910)	Patients without SCT (*n* = 4503)	Total patients (*N* = 6413)
Age (years) at index date			
Mean (SD)	58 (8.1)	73.5 (9.71)	68.9 (11.67)
Median (range)	60 (26–79)	74 (22–99)	69 (22–99)
IQR	11	13	17
Age group (years) at index date (%)			
18–30	4 (0.2)	4 (0.1)	8 (0.1)
31–50	334 (17.5)	121 (2.7)	455 (7.1)
51–70	1540 (80.6)	1445 (32.1)	2985 (46.5)
> 70	32 (1.7)	2933 (65.1)	2965 (46.2)
Sex (%)			
Male	1070 (56.0)	2273 (50.5)	3343 (52.1)
Female	840 (43.9)	2230 (49.5)	3070 (47.9)
Charlson score adapted to SNDS			
Mean (SD)	2.3 (0.61)	2.8 (1.2)	2.6 (1.1)
Median (range)	2 (2–7)	2 (2–10)	2 (2–10)
IQR	0	1	1
Charlson score per class adapted to SNDS (%)			
0	–	–	–
1–2	1559 (81.6)	2578 (57.3)	4137 (64.5)
3–4	325 (17.0)	1447 (32.1)	1772 (27.6)
≥ 5	26 (1.4)	478 (10.6)	504 (7.9)
Number of LOT per patient during follow-up (%)			
Undetermined	27 (1.4)	157 (3.5)	184 (2.9)
1	663 (34.7)	1891 (42.0)	2554 (39.8)
2	565 (29.6)	1214 (27.0)	1779 (27.7)
3	306 (16.0)	604 (13.4)	910 (14.2)
4	129 (6.8)	278 (6.2)	407 (6.3)
5 +	220 (11.5)	359 (8.0)	579 (9.0)
Follow-up duration, months (%)			
Mean (SD)	33.2 (19.0)	23.1 (18.5)	26.1 (19.2)
Median (range)	30 (4–72)	19 (0–72)	22 (0–72)
IQR	31	28	29
Reason for end of follow-up (%)			
Death	279 (14.6)	1888 (41.9)	2167 (33.8)
Disenrollment^a^	4 (0.2)	2 (0.04)	6 (0.1)
Lost to follow-up^a^	4 (0.2)	19 (0.4)	23 (0.4)
End of observation period	1623 (85.0)	2594 (57.6)	4217 (65.8)

^a^Defined as cases with a gap of 12 months without claims registered in the SNDS for which no death was registered

*IQR* interquartile range, *LOT* line of treatment, *SCT* stem cell transplant, *SD* standard deviation, *SNDS* Système National des Données de Santé

The median Charlson comorbidity score for the study cohort was 2 (IQR, 1). The most common comorbidities were diabetes (*n* = 1065; 16.6%), moderate or severe renal disease (*n* = 1035; 16.1%), and chronic pulmonary disease (*n* = 809; 12.6%) (Supplementary Table 5). The number of patients was evenly distributed across the study inclusion period.

In total, 1910 patients (29.8%) received SCT, 97.7% (*n* = 1866) of which was autologous SCT. The majority (98%) of patients received their first transplant within the first year of follow-up. Patients in the SCT subgroup were younger and had fewer comorbidities than patients without SCT (median age, 60 vs 74 years, and 81.6% vs 57.6% with Charlson comorbidity score < 3, respectively) (Table 1). Median follow-up for patients with SCT was longer than for those without SCT (30 vs 19 months, respectively), with a smaller proportion of patients lost to follow-up because of death (14.6% vs 41.9%, respectively).

Overall, 2554 patients (39.8%) received a single LOT during follow-up, and 579 patients (9%) received at least 5 LOTs (Table 1; Supplementary Table 6). Fewer patients with SCT received only 1 LOT compared with those without SCT (34.7% vs 42.0%, respectively), and fewer patients who received SCT died compared with those who did not (14.6% vs 41.9%, respectively).

### Treatment regimens used across lines of therapy by transplantation status

**Fig. 2:**
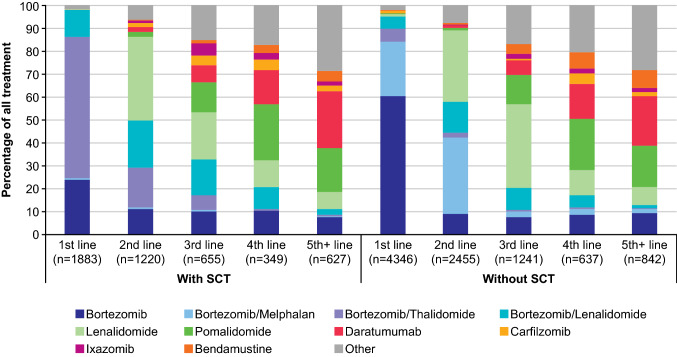
Treatment regimens used across LOTs by SCT status. Dexamethasone and prednisone were not always observed (owing to incomplete data availability), so for some patients use in combination with other treatments is assumed. *LOT* line of treatment, *SCT *stem cell transplant

Almost all the study cohort received treatment with an identifiable drug during follow-up (*n* = 6229; 97.1%). Patients without an identifiable drug (*n* = 184; 2.9%) were referred to as undetermined LOT (Table 1). The most frequently administered drug regimens based on treatment guidelines are presented by LOT and SCT status in Fig. 2 and Supplementary Table 7.

Overall, bortezomib-based regimens were the most commonly prescribed regimen (*n* = 8865, 62.2%), with 6026 patients (96.7%) receiving this combination at LOT1. Lenalidomide-based regimens were the most frequently administered regimen at LOT2 (*n* = 1213, 17%) and LOT3 (583, 15%). Treatment choice was more diverse for later LOTs, with 37 distinct regimens identified at LOT4 + . Regimens based on lenalidomide, pomalidomide, or daratumumab were most frequently administered at later LOTs. Of note, dexamethasone and prednisone were only partially observed in the database and use in combination with the other treatments was assumed for some patients. The median duration of treatment decreased with each subsequent LOT from 9.3 months in SCT recipients (5.6 in non-SCT recipients) for LOT1 to approximately 2 months for LOT5 + (Supplementary Fig. 3, Supplementary Table 8).

### All-cause HCRU and associated costs

Total MM HCRU cost during the study was €816 million (M), with more than half (€464 M) accrued during the first year following MM diagnosis (Table 2). The mean annual cost per patient was €58.3 thousand (K), and the bulk of this cost was attributed to treatment (€28.2 K) and hospitalisation (€22.2 K). The mean total annual cost per patient in the first year exceeded €72.4 K, with the monthly cost more than €7.1 K. Almost all patients in the study cohort (*n* = 6194, 96.6%) underwent some type of all-cause hospitalisation during follow-up, including 5968 patients (93.1%) who experienced at least one overnight stay in hospital (Table 2). The overall rate was 6.3 hospitalisations PPPY. Hospitalisations accounted for a greater proportion of total cost in the first year (48.6%) than the average of all years analysed (38.1%) (€35.2 K of €72.4 K total vs €48.5 K of €127.2 K total, respectively). Of €311.1 M total hospitalisation cost, €225.6 M was accrued in the first year.

**Table 2:** All-cause HCRU and costs for the overall MM population (*N* = 6413)

HCRU	Total cost			Annual cost	1st year cost			Monthly cost in 1st year
	Sum, M€	Mean per person, K€	%	PPPY, K€	Sum, M€	Mean, K€	%	PPPM, K€
Hospitalisations excl. chemotherapies	206.14	32.14	25.3	14.74	145.17	22.64	31.3	2.23
Chemotherapy sessions	71.84	11.20	8.8	5.14	54.69	8.53	11.8	0.84
Hospitalisations at home	14.62	2.28	1.8	1.05	11.09	1.73	2.4	0.17
Hospitalisation in rehabilitation centre	18.40	2.87	2.3	1.32	14.65	2.28	3.2	0.23
Emergency stays	0.10	0.02	0.0	0.01	0.05	0.01	0.0	0.00
All hospitalisation	311.10	48.51	38.1	22.24	225.64	35.19	48.6	3.47
“High-cost drugs”^1^ including:	137.17	21.39	16.8	9.81	114.09	17.79	24.6	1.76
Bortezomib	119.80	18.68	14.7	8.56	106.28	16.57	22.9	1.64
Bendamustine	0.83	0.13	0.1	0.06	0.22	0.03	0.0	0.00
Carfilzomib	2.91	0.45	0.4	0.21	0.21	0.03	0.0	0.00
Doxorubicine	0.26	0.04	0.0	0.02	0.17	0.03	0.0	0.00
“Temporary Authorized drugs”^2^ including	42.04	6.56	5.2	3.01	4.92	0.77	1.1	0.08
Daratumumab	41.43	6.46	5.1	2.96	4.63	0.72	1.0	0.07
“Retrocession drugs”^3^ including:	163.92	25.56	20.1	11.72	38.15	5.95	8.2	0.59
Lenalidomide	109.02	17.00	13.4	7.79	26.79	4.18	5.8	0.41
Dexamethasone	2.80	0.44	0.3	0.20	1.32	0.21	0.3	0.02
Thalidomide	4.71	0.73	0.6	0.34	4.42	0.69	1.0	0.07
Pomalidomide	41.70	6.50	5.1	2.98	3.44	0.54	0.7	0.05
“Drugs in community”^4^ including:	51.76	8.07	6.3	3.70	26.04	4.06	5.6	0.40
Melphalan	0.94	0.15	0.1	0.07	0.88	0.14	0.2	0.01
Ixazomib	0.80	0.13	0.1	0.06	0.06	0.01	0.0	0.00
All treatment^a^	394.89	61.58	48.4	28.23	183.21	28.57	39.5	2.82
Laboratory tests	12.61	1.97	1.5	0.90	6.00	0.94	1.3	0.09
Medical procedures	8.93	1.39	1.1	0.64	4.50	0.70	1.0	0.07
Physician visits^b^	5.82	0.91	0.7	0.42	2.38	0.37	0.5	0.04
Other health professional visits^c^	18.90	2.95	2.3	1.35	8.44	1.32	1.8	0.13
Transport	31.51	4.91	3.9	2.25	18.91	2.95	4.1	0.29
Medical devices	10.44	1.63	1.3	0.75	4.90	0.76	1.1	0.08
Sick leave & invalidity	21.79	3.40	2.7	1.56	10.10	1.58	2.2	0.16
All other costs	110.00	17.15	13.5	7.86	55.23	8.61	11.9	0.85
Total	816.00	127.24	100	58.34	464.09	72.37	100	7.14

^a^Includes sum of ^1–4^, ^b^GP and specialist, ^c^Nurse, physiotherapist, and other

*GP* general practitioner, *HCRU* health care resource utilisation, *K* thousands, *M* millions, *MM* multiple myeloma, *PPPM* per person per month analysis, *PPPY* per person per year

Among treatment costs during follow-up, retrocession drugs accrued the highest cost (€11.72 K PPPY, including €5.95 K during the first year), but the “high-cost drugs” contributed the greatest cost during the first year (€17.8 K PPPY). Among the “high-cost drugs”, bortezomib cost €8.56 K PPPY, while the “retrocession drug” lenalidomide cost €7.79 K PPPY (Table 2). Almost all patients (6241 of 6413 total patients, 97.3%) received “high-cost drugs”, where the “high cost” was linked to the use of bortezomib-based regimens in LOT1 (data not shown).

In general, HCRU was lower for patients with SCT compared with total population (Table 3). While the average duration of hospital stays was similar between groups (11.1 days for SCT sub-group compared with 11.8 days in no SCT), hospitalisations were less frequent among patients with SCT compared with total patient population (4.7 vs 6.3 per PPPY) but accrued higher costs. This was largely attributable to the more expensive SCT procedure (€24 K per transplant vs €0.9 K for average medicine, surgery, or obstetrics hospitalisation). The rate of sick-leave payment was also much greater for those with SCT compared with the total population (64.4 vs 28.5 days PPPY, respectively), as were rates of laboratory tests and medical procedures.

**Table 3:** HCRU per person per year (in days) by LOT

HCRU	LOT1 (*n* = 6229)		LOT2 (*n* = 3675)		LOT3 (*n* = 1896)		LOT4 (*n* = 986)		LOT5 + (*n* = 579)		Patients with SCT (*n* = 1910)		Total (*N* = 6413)	
	*n* (%)	PPPY	*n* (%)	PPPY	*n* (%)	PPPY	*n* (%)	PPPY	*n* (%)	PPPY	*n* (%)	PPPY	*n* (%)	PPPY
Total MCO hospitalisations	4692 (75.3)	6.3	2327 (37.4)	5.6	1169 (18.8)	6.1	582 (9.3)	7.3	476 (7.6)	10.3	1910 (100.0)	4.7	6194 (96.6)	6.3
Complete MCO hospitalisations	4002 (64.2)	1.9	1834 (29.4)	1.3	906 (14.5)	1.5	429 (6.9)	2.0	411 (6.6)	2.5	1895 (99.2)	1.6	5968 (93.1)	1.9
Chemotherapy sessions	5893 (94.6)	19.3	2487 (39.9)	10.9	970 (15.6)	8.7	568 (9.1)	11.8	433 (7.0)	14.8	1901 (99.5)	22.1	6199 (96.7)	18.9
Hospitalisations at home	857 (13.8)	1.7	325 (5.2)	1.0	106 (1.7)	0.4	64 (1.0)	0.9	67 (1.1)	0.7	392 (20.5)	0.7	1159 (18.1)	0.9
Hospitalisation in rehabilitation centre	1033 (16.6)	0.3	283 (4.5)	0.1	144 (2.3)	0.2	61 (1.0)	0.2	60 (1.0)	0.2	590 (30.9)	0.1	3096 (48.3)	0.2
Emergency stays	1071 (17.2)	0.3	632 (10.1)	0.3	259 (4.2)	0.3	104 (1.7)	0.3	102 (1.6)	0.3	759 (39.7)	0.2	2360 (36.8)	0.3
GP physician visits	5055 (81.2)	8.8	3068 (49.3)	9.1	1378 (22.1)	8.8	678 (10.9)	8.7	460 (7.4)	8.6	1862 (97.5)	8.5	5959 (92.9)	8.7
Specialist physician visits	4429 (71.1)	5.7	2957 (47.5)	6.7	1357 (21.8)	7.3	629 (10.1)	6.2	412 (6.6)	6.2	1889 (98.9)	6.1	5671 (88.4)	6.1
Transport	5309 (85.2)	22.7	2837 (45.5)	17.3	1360 (21.8)	15.9	813 (13.1)	18.9	813 (13.1)	25.2	1789 (93.7)	14.1	5885 (91.8)	17.4
High-cost medical devices	184 (3.0)	0.2	92 (1.5)	0.1	28 (0.4)	0.1	17 (0.3)	0.1	11 (0.2)	0.1	129 (6.8)	0.1	494 (7.7)	0.2
Sick leavea & invalidity	836 (13.4)	56.0	259 (4.2)	14.3	112 (1.8)		56 (0.9)		55 (0.9)		764 (40.0)	64.4	989 (15.4)	28.5

^a^*n * number of days

*GP* general practitioner, *HCRU* health care resource utilisation, *LOT* line of treatment, *MCO* medicine, surgery, and obstetrics, *PPPY* per person per year, *SCT* stem cell transplant

When evaluating the type of HCRU utilized by LOT, hospitalisation rates declined from LOTs 1–4, but increased in the LOT5 + group (Supplementary Table 9). As expected, ambulatory chemotherapy sessions were most common in LOT1 (92.2%) and were favoured over hospitalisations for chemotherapy (22.0%).

### AE-related HCRU and associated costs

In total, 2901 patients (45.2%) had an event of interest at primary diagnosis of MM. The most commonly reported were infections that were not otherwise specified (*n* = 1200; 18.7%) (Supplementary Fig. 4). During follow-up, there were 7397 hospitalisations due to events of interest associated with MM (4463 within the first year after diagnosis), at the total cost of €29.6 M (9.5% of all hospitalisation costs) (Supplementary Table 10). The share of hospitalisation cost due to an event of interest was lower (8.0%) in patients with SCT than the total study cohort, but the monthly rate in the first year was the same (€0.28 K PPPM). The proportional cost of AE hospitalisations increased with subsequent LOTs from 8.5% of total cost of hospitalisations at LOT1 to over 14.6% of all hospitalisations during LOT5 and beyond.

### HCRU related to the administration of MM treatment only and associated costs

The sum resource cost of MM-related treatment administration was €412 M (50.5% of the total HCRU cost for the study cohort), and during the first year following diagnosis the cost was €3.3 K PPPM (Table 4). The table provides a breakdown of drugs by categories; “high-cost drugs” which includes bortezomib, “temporarily authorised drugs” which includes daratumumab and “retrocession drugs” which includes lenalidomide, pomalidomide, and thalidomide (Supplementary Table 1). The largest component of the cost was treatment with “retrocession” and “high-cost” drugs. This is consistent with the high costs associated with lenalidomide and bortezomib (€7.79 K and €8.56 K PPPY, respectively; Table 2) reflected in the all-cost HCRU analysis. However, as noted above, regimens based on lenalidomide, pomalidomide, or daratumumab were most frequently administered at later LOTs. It is important to note that lenalidomide was removed from the “high-cost drugs” category in 2013 and daratumumab wasn’t added to “high-cost drugs” category until after the end of this study period. Monthly costs of MM-related treatment administration per patient in the first year in patients with SCT were similar to the total population (€3.314 K and €3.302 K, respectively). The monthly cost of MM-related treatment administration per patient in the first year increased with each subsequent LOT from €2.447 K in LOT1 to €7.026 K in LOT5 + . This increase could largely be attributed to greater use of “temporarily authorised” and “retrocession drugs” between LOT1 and later LOTs.

**Table 4:** Costs of MM-related treatment administration by LOT

	LOT1 (*n* = 6229)	LOT2 (*n* = 3675)	LOT3 (*n* = 1896)	LOT4 (*n* = 986)	LOT5 + (*n* = 579)	Patients with SCT (*n* = 1910)	Total (*N* = 6413)
Mean resource cost, K€							
Chemotherapy sessions (related to MM diagnosis)	6.43	3.88	2.31	2.46	5.75	13.74	10.72
MM “high-cost drugs”^a^	12.94	6.47	2.65	2.86	5.91	20.28	19.19
MM “temporarily authorised drugs”^b^	0.09	0.90	4.75	10.20	27.61	9.82	6.49
MM “retrocession drugs”^c^	2.47	17.75	22.37	16.28	28.19	32.21	24.73
MM “drugs in community”	0.09	0.13	0.22	0.15	0.22	0.23	0.29
MM treatment	15.60	25.26	30.00	29.48	61.93	62.54	50.71
Transport (following chemotherapy)	1.11	0.70	0.37	0.42	1.13	1.99	1.83
Sick leave (7 days following chemotherapy)	0.60	0.30	0.26	0.19	0.72	2.80	0.98
Total cost treatment related MM	23.75	30.15	32.94	32.56	69.53	81.07	64.25
Share of treatment related MM in total cost	48%	61%	66%	69%	69%	49%	50%
Sum resource cost, M€							
Chemotherapy sessions (related to MM diagnosis)	40.07	14.28	4.39	2.42	3.33	26.24	68.77
MM “high-cost drugs”^a^	80.62	23.78	5.02	2.82	3.42	38.73	123.09
MM “temporarily authorised drugs”^b^	0.58	3.30	9.01	10.05	15.99	18.75	41.65
MM “retrocession drugs”^c^	15.40	65.25	42.42	16.05	16.32	61.25	158.62
MM “drugs in community”	0.59	0.49	0.42	0.15	0.13	0.45	1.88
MM treatment	97.20	92.82	56.88	29.07	35.86	119.45	352.23
Transport (following chemotherapy)	6.93	2.58	0.69	0.42	0.65	3.81	11.75
Sick leave (7 days following chemotherapy)	3.75	1.12	0.49	0.19	0.42	5.34	6.26
Total cost treatment related MM	147.96	110.80	62.45	32.10	40.26	154.84	412.01
Monthly resource cost in the first year, K€							
Chemotherapy sessions (related to MM diagnosis)	0.663	0.367	0.307	0.454	0.581	0.852	0.812
MM “high-cost drugs”^a^	1.334	0.612	0.351	0.528	0.597	1.435	1.636
MM temporarily “authorised drugs”^b^	0.010	0.085	0.631	1.883	2.790	0.053	0.072
MM “retrocession drugs”^c^	0.255	1.679	2.968	3.007	2.849	0.679	0.556
MM “drugs in community”	0.010	0.013	0.030	0.027	0.022	0.001	0.016
MM treatment	1.608	2.389	3.980	5.446	6.258	2.168	2.279
Transport (following chemotherapy)	0.115	0.066	0.049	0.078	0.114	0.121	0.140
Sick leave (7 days following chemotherapy)	0.062	0.029	0.034	0.035	0.073	0.172	0.071
Total cost treatment related MM	2.447	2.852	4.369	6.014	7.026	3.314	3.302

^a^Includes bortezomib, ^b^Includes daratumumab, ^c^Includes lenalidomide, pomalidomide, and thalidomide

*K* thousands, *LOT* line of treatment, *M* millions, *MM* multiple myeloma, *SCT* stem cell transplant

### HCRU related only to MM and associated costs

During follow-up, the cost of HCRU related to MM specifically was €611.5 M (74.9% of the total HCRU cost), with over half of the cost accrued within the first year after MM diagnosis (annual rate of €367.11 K per patient) (Supplementary Table 11). Costs per patient increased with later LOTs, more than doubling from €3.9 K PPPM at LOT1 to €8.5 K PPPM at LOT5 + , mainly attributable to the increased cost of drugs between LOT1 and LOT5 + . However, fewer patients received later lines of treatment (LOT1 *n* = 6229 vs LOT5 + *n* = 579).

## Discussion

This analysis of a comprehensive HCRU database with national coverage in France demonstrates that MM represents a substantial economic burden to health care systems. For patients diagnosed between 2013 and 2018 receiving active treatment, the overall cost in France of treating MM was estimated to be €58.3 K PPPY, with more than half of the costs accrued in the first year after diagnosis. The greatest costs were attributed to treatment and hospitalisation. This study extends findings of earlier research in Europe that reported lower HCRU costs for MM, but most used smaller cohorts or modelled data [14, 16, 26, 27]. In one study reporting MM costs for 2018 in Portugal, the average overall annual MM cost burden per patient was lower (€31 K PPPY) than reported here (€58.3 K PPPY) [27]. Yet, the difference in the average annual cost of treatment administration was smaller (€28 K PPPY reported here vs €25 K PPPY reported in Portugal) and both studies show that the cost were mainly driven by the hospitalisations and treatments [27]. However, the Portuguese study was based on a lower number of patients than the current study (*n* = 1941 vs *n* = 6413) and did not take into account costs associated with sick leave and invalidity. Another French study looking at MM HCRU costs in patients who received at least 1 prior treatment before the period 2004–2005 estimated monthly costs of treatments to be €2.1 K (vs €2.8 K reported here) [15].

The higher costs reported in this study reflect directly reported HCRU from a nationwide database and are likely to be a more representative estimate of the burden of MM in France [16, 28]. Although the current results are consistent with the recent French Health Insurance Fund report, the higher global costs found in the current study may be due to differences in the studied populations: the current study included actively treated incident patients (2013–2018), whereas the previous report evaluated costs in the prevalent population [13]. Furthermore, the greater hospitalisation costs in the current analysis were found to be higher in the first year of follow-up, which likely is due to a difference between an incident population vs a prevalent population [13]. Approximately 30% of patients underwent SCT, consistent with recently published data [29, 30]; these patients accounted for greater financial impact, particularly in the first year after diagnosis, than the average for the total population. They were generally younger, in line with published data [30], and experienced fewer hospitalisations during follow-up than the total population in a manner that was constant over time. The expensive SCT procedure contributed to higher hospitalisation costs. Extended hospitalisation required for SCT and the younger age of these patients may also have contributed to increased costs related to sick leave.

In France, as in the broader global context [31], no regimen is currently recommended as a standard of care for heavily pretreated and relapsed MM. Consistent with previous findings [29], LOT1 treatment was quite uniform during the study period (with 97% of patients receiving bortezomib-based regimens). However, published global data showed lower use of bortezomib based regimens in non-SCT population than in this study (54% vs 96%, respectively) [30]. Treatment diversity in later LOTs, reflected earlier real-word evidence [30], was associated with a substantial increase in patient costs. Monthly costs related to MM for patients who received LOT5 + were twice those for patients who received up to 2 LOTs, presumably due to more severe disease and often higher costs of newer therapies; earlier studies have also indicated that later LOTs may be more expensive [15, 18]. In the current analysis, use of retrocession drugs such as immunomodulatory drugs (especially pomalidomide) at LOT3 was a major contributor to increased cost of later treatment regimens, consistent with practice reported in other European regions [16, 17]. However, drugs such as pomalidomide, lenalidomide, and daratumumab are increasingly used in earlier treatment regimens until disease progression [32, 33]. These treatment indication changes will impact the economic burden of MM. The facility for newer therapies, such as daratumumab, to be recorded during the follow-up period means that this study captured more complete drug costs and hospitalisations data than earlier studies, including a multi-country European study which did not cover treatments approved post-2015 [16]. Of note, the price of daratumumab in the French system has decreased since the analysis of this database, so this may contribute to increased use of this drug in the future [34].

Increase in hospitalisation costs associated with subsequent LOT was likely related to the increasing age and decreasing health of patients requiring ongoing treatment for MM. This is supported by a retrospective study of hospitalised French patients, which identified an association between age and duration of hospital stay [35]. The cost of hospitalisations for an event of interest constituted a greater proportion of hospitalisation costs among patients at later LOT, indicating that declining patient condition conferred a greater burden to the health care system beyond the cost of drugs. Ultimately, more effective treatments are needed to avoid multiple LOTs and reduce the economic burden of MM.

### Strengths

Unlike several published case review studies, this study analysed data from the SNDS, a large comprehensive database with national coverage of health care costs throughout France, which allows most patients to be studied from birth to death. Although the database allowed to identify a cohort of newly diagnosed patients with MM, with no patients excluded based on demographic or health status [36], because of the stringent inclusion criteria the sample may not be representative of all newly diagnosed patients. Previous European studies used model estimates to analyse HCRU costs (e.g., a Dutch study of patients treated up to LOT3 [28]), the costs reported in this study came from a database of actual costs accrued, representing an accurate estimate for routine clinical care for MM in both inpatient and outpatient settings in France. Furthermore, this study assessed patients up to LOT5 + , allowing the substantial increase in per patient costs in later LOTs to be fully characterised. To account for the absence of clinical or paraclinical test results in the database, a validated algorithmic definition of LOT allowed accurate identification of LOT for this large cohort, despite missing details on combination therapies [23]. This analysis not only provided all-cause HCRU for patients with incident MM but also confirmed the costs specifically associated with an MM diagnosis.

## Limitations

Patients with pre-existing MM, those with a single record of MM, as well as those treated later than 30 days after the initial diagnosis were excluded. This approach was undertaken to increase the precision of the MM-related costs; however, in so doing, the size of the study cohort was substantially reduced, potentially excluding clinically eligible patients. In addition, the focus on actively treated patients may have skewed the results towards higher cost patients and excluded those for whom early treatment was not possible, e.g., due to underlying complexities. Therefore, the stringent eligibility criteria and the inherent lack of clinical and pathological information in health insurance databases may have reduced ability to accurately identify all patients with newly diagnosed MM. Furthermore, although our study was designed to limit the included patients to only those with newly diagnosed MM, there is the possibility that some patients with RRMM may have been miscoded or mistakenly included in the sample.

As not all drugs were observable or fully observable in the database, the LOT was derived algorithmically, and some assumptions may not be accurate for a small proportion of LOT definitions. Although the algorithm is robust in identifying LOT and its duration, it cannot provide the granular detail of all the components of the regimen, since not all regimens are observable in SNDS database. Consequently, the algorithm is not precise enough for analysing specific regimens (combination of drugs); for example, the 40-day overlap criterion may not be optimal for some regimens at LOT3 + . Inclusion of some drugs that were only partially observable (melphalan and cyclophosphamide) may have resulted in overestimation of the number of LOT per patient identified by the algorithm and underestimation of LOT1 treatment duration. This may especially apply to the number of patients without SCT who received bortezomib/melphalan in LOT2. In addition, the lack of care plans availability in the database means that for some patients some drugs may have been used in combination with other treatments without being observed in the database. Therefore, the results should be interpreted with caution because true HCRU costs may have been underestimated. The study design introduced a follow-up time bias because patients who died before receiving a transplant were included in the non-SCT rather than the SCT subgroup in the database, leading to patients with a short follow-up being recorded as non-SCT patients.

Fewer patients who received later LOTs than those who received LOT1 were captured in the study, consistent with previous findings [29]. Thus the relatively short period of the study may have not fully captured the economic impact of SCT as patients who undergo the SCT are younger, live longer, and are more likely to need multiple LOT.

## Conclusions

This comprehensive analysis of the SNDS database demonstrates high HCRU and costs associated with treating MM in France and high variation in treatment patterns for late-line treatment strategy.

These data provide a valuable, up-to-date resource to inform stakeholders around healthcare costs in MM and demonstrate the significant disease burden in this patient population. The high HCRU and cost reflect the high MM burden in France. In the future, as more treatments become available, the cost of MM treatment is expected to grow further, especially if the incidence of MM continues to rise. Therefore, development of effective and safe new treatments is critical to help mitigate those costs. Further studies would be needed to improve the treatment-defining algorithm and to accurately analyse the treatment patterns of MM patients.

## Supplementary Information

Below is the link to the electronic supplementary material.

Supplementary file1 (EPS 2204 KB)

Supplementary file2 (EPS 1689 KB)

Supplementary file3 (EPS 1194 KB)

Supplementary file4 (EPS 1309 KB)

Supplementary file5 (EPS 1617 KB)

Supplementary file6 (DOCX 126 KB)

## Data Availability

GlaxoSmithKline makes available anonymised individual participant data upon approval of proposals submitted to www.clinicalstudydatarequest.com. To access data for other types of GlaxoSmithKline sponsored research, for study documents without patient-level data, and for clinical studies not listed, please submit an enquiry via the website.
