# Supplementary material for: Assessing the treatment pattern, health care resource utilisation, and economic burden of multiple myeloma in France using the Système National des Données de Santé (SNDS) database: a retrospective cohort study
Source: Eur J Health Econ. 2022 May 25;24(3):321–33. doi: 10.1007/s10198-022-01463-9 (PMC10060291; doi:10.1007/s10198-022-01463-9)
Supplement: Supplementary file 6 — Supplementary file6 (DOCX 126 KB) [file 10198_2022_1463_MOESM6_ESM.docx]

# Supplementary Materials

## Supplementary Figure 1. An overview of the algorithm used to define sequential LOT with examples

Figure adapted with permission from Palmaro et al 2017.[[23](#_ENREF_23)] A LOT was defined as continuing until a new drug was added (excluding widely used therapies such as corticosteroids). If a new regimen was prescribed before the previous LOT was completed, the new LOT was considered a combination treatment when the overlap was longer than 40 days; if the overlap was less than 40 days, the drug with the longest duration of treatment was identified as the regimen.
LOT, line of treatment; Rd, lenalidomide plus dexamethasone.

## Supplementary Figure 2. Study attrition

ICD-10, International Classification of Diseases version 10; MM, multiple myeloma; SNDS, Système National des Données de Santé.

## Supplementary Figure 3. Treatment duration by LOT by SCT status

1. Patients who received SCT
2. Patients who did not receive SCT

LOT, line of treatment; SCT, stem cell transplant.

## Supplementary Figure 4. AEs at primary diagnosis showing proportion of whole study cohort affected

AE, adverse events; GI, gastrointestinal.

## Supplementary Table 1. The category and observability of different classes of MM drugs in SNDS

| **Class** | **Molecule** | **Drug category** | | | | **Drugs fully observable in SNDS** | **Drugs used to identify LOT** |
| --- | --- | --- | --- | --- | --- | --- | --- |
|  |  | **“High-cost drugs”** | **“Temporarily authorised drugs”/**  **Post-“temporarily authorised drugs”** | **“Retrocession drugs”** | **“Drugs in community”** |  |  |
| Proteasome inhibitors | Bortezomib | X |  |  |  | X | X |
|  | Carfilzomib | X | X |  |  | X | X |
|  | Ixazomib |  |  | X | X | (X) | X |
| Immunomodulatory imide drugs | Lenalidomide^a^ |  |  | X |  | X | X |
|  | Pomalidomide |  |  | X |  | X | X |
|  | Thalidomide |  |  | X |  | X | X |
| Monoclonal antibody | Daratumumab^b^ |  | X |  |  | (X) | X |
| Alkylating agents | Melphalan |  |  |  | X |  | X |
|  | Cyclophosphamide |  |  |  | X |  | X |
|  | Bendamustine^c^ | X |  |  |  | X | X |
| Topoisomerase inhibitors | Doxorubicin | X |  |  |  | X | X |
| Corticosteroids | Dexamethasone |  | X | X | X |  |  |
|  | Prednisone |  |  |  | X |  |  |

^a^Removed from “high-cost drugs” in 2013; ^b^Daratumumab was added to “high-cost drugs” following the end of the study period; ^c^Partially removed from “high-cost drugs” during study period. (X) denotes incomplete observability for some years during the study period.
LOT, line of treatment; MM, multiple myeloma; SNDS, Système National des Données de Santé database.

## Supplementary Table 2. Stem cell transplant diagnosis-related group codes and Common Classification of Medical Procedures code

| **Diagnosis-related group ^a^** | **Code** |
| --- | --- |
| Allogeneic stem cell transplant | 27Z03Z |
| Allogeneic stem cell transplant: level 1 | 27Z021 |
| Allogeneic stem cell transplant: level 2 | 27Z022 |
| Allogeneic stem cell transplant: level 3 | 27Z023 |
| Allogeneic stem cell transplant: level 4 | 27Z024 |
| Stem cell transplantation: outpatient | 27Z04J |
| **CCAM code** |  |
| IV injection of a cellular therapy product for allogeneic cell transplant | FELF009 |

^a^Referred to as ‘Groupe homogène de maladies (GHM)’ code in SNDS database.
CCAM, Common Classification of Medical Procedures (Classification Commune des Actes Médicaux); IV; intravenous injection.

## Supplementary Table 3. ICD-10 codes used for the identification of AEs of interest

| **ICD-10 Code** | **Description** |
| --- | --- |
| **Keratopathy/keratitis** | |
| H160 | Corneal ulcer (incuding NOS, central, marginal, perforated, ring, with hypopyon, Mooren) |
| H161 | Other superficial keratitis without conjunctivitis (inc. areolar, filamentary, nummular, stellate, striate, superficial punctate, photokeratitis, snow blindness) |
| H162 | Keratoconjunctivitis (NOS, exposure, neurotrophic, phlyctenular, ophthalmia nodosa, superficial keratitis with conjunctivitis) |
| H163 | Interstitial and deep keratitis |
| H164 | Corneal neovascularization (Ghost vessels, pannus) |
| H168 | Other keratitis |
| H169 | Keratitis, unspecified |
| **Cataracts** | |
| H263 | Drug-induced cataract |
| H269 | Cataract, unspecified |
| **Glaucoma** | |
| H400 | Glaucoma suspect |
| H401 | Primary open-angle glaucoma |
| H402 | Primary angle-closure glaucoma |
| H406 | Glaucoma secondary to drugs |
| H408 | Other glaucoma |
| H409 | Glaucoma, unspecified |
| **Upper GI bleeding** | |
| K920 | Haematemesis |
| K921 | Melaena |
| I850 | Oesophageal varices with bleeding |
| I983 | Oesophageal varices with bleeding in disease classified elsewhere |
| K2210 | Ulcer of oesophagus, acute with bleeding |
| K2212 | Ulcer of oesophagus, acute with both bleeding and perforation |
| K2214 | Ulcer of oesophagus, chronic or unspecified with bleeding |
| K2216 | Ulcer of oesophagus, chronic or unspecified with both bleeding and perforation |
| K250 | Gastric ulcer, acute with bleeding |
| K252 | Gastric ulcer, acute with both bleeding and perforation |
| K254 | Gastric ulcer, chronic or unspecified with bleeding |
| K256 | Gastric ulcer, chronic or unspecified with both bleeding and perforation, |
| K260 | Duodenal ulcer, acute with bleeding |
| K262 | Duodenal ulcer, acute with both bleeding and perforation |
| K264 | Duodenal ulcer, chronic or unspecified with bleeding |
| K266 | Duodenal ulcer, chronic or unspecified with both bleeding and perforation |
| K270 | Peptic ulcer, acute with bleeding |
| K262 | Duodenal ulcer, acute with both bleeding and perforation |
| K264 | Duodenal ulcer, chronic or unspecified with bleeding |
| K266 | Duodenal ulcer, chronic or unspecified with both bleeding and perforation |
| K270 | Peptic ulcer, acute with bleeding |
| K272 | Peptic ulcer, acute with both bleeding and perforation |
| K274 | Peptic ulcer, chronic or unspecified with bleeding |
| K276 | Peptic ulcer, chronic or unspecified with both bleeding and perforation |
| K280 | Gastrojejunal ulcer, acute with bleeding |
| K282 | Gastrojejunal ulcer, acute with both bleeding and perforation |
| K284 | Gastrojejunal ulcer, chronic or unspecified with bleeding |
| K286 | Gastrojejunal ulcer, chronic or unspecified with both bleeding and perforation |
| K290 | Acute bleeding gastritis |
| K6380 | Angiodysplasia of small intestine, except duodenum with bleeding |
| K3180 | Angiodysplasia of stomach and duodenum with bleeding |
| **Lower GI bleeding** | |
| K552 | Angiodysplasia of colon with bleeding |
| K625 | Bleeding of anus and rectum |
| K922 | Gastrointestinal bleeding, unspecified |
| **Intracranial** **haemorrhage** | |
| I60 | Subarachnoid haemorrhage |
| I61 | Intracerebral haemorrhage |
| I620 | Subdural haemorrhage |
| I621 | Nontraumatic extradural haemorrhage |
| I629 | Intracranial, nontraumatic, unspecified haemorrhage |
| **Other major bleeding** | |
| N02x | Recurrent and persistent haematuria |
| K661 | Hemoperitoneum |
| N938 | Other specified abnormal uterine and vaginal bleeding |
| N939 | Abnormal uterine and vaginal bleeding, unspecified |
| N950 | Postmenopausal bleeding |
| R041 | Bleeding from throat |
| R042 | Haemoptysis |
| R048 | Bleeding from other sites in respiratory passages |
| R049 | Bleeding from respiratory passages, unspecified |
| R310 | Gross hematuria |
| R311 | Microscopic hematuria |
| R318 | Other and unspecified hematuria |
| R58 | Bleeding, not elsewhere classified |
| D683 | Haemorrhagic disorder due to circulating anticoagulants |
| H356 | Retinal bleeding |
| H431 | Vitreous bleeding |
| H450 | Vitreous bleeding in diseases classified elsewhere |
| M250 | Haemarthrosis |
| **Infusion reaction** | |
| T80x | Complications following infusion, transfusion and therapeutic injection |
| **Dry eye** | |
| H041 | Other disorders of lacrimal gland |
| **Light sensitivity (photophobia)** | |
| H531 | Subjective visual disturbances (asthenopia, day blindness, hemeralopia, metamorphosia, photophobia, scintillating scotoma, sudden visual loss and visual halos) |
| **Blurred vision** | |
| H538 | Other visual disturbances |
| **Anaemia** | |
| D59x | Acquired hemolytic anaemia |
| D60x | Acquired pure red cell aplasia [erythroblastopenia] |
| D61x | Other aplastic anaemias |
| D63x | Anemia in chronic diseases classified elsewhere |
| D64x | Other anaemias |
| **Neutropenia** | |
| D70x | Agranulocytosis |
| **Trombocytopenia** | |
| D695 | Secondary thrombocytopenia |
| **Infection** | |
| A00x-B99x | Certain infectious and parasitic diseases |
| G00‐G07, G530, G531, G630, G734, G940 | Nervous system infections |
| H00, H050, H061, H100, H130, H131, H190‐H192, H220, H320, H440, H441, H451, H588, H598 | Ophthalmological infections |
| H600‐H603, H620‐H624, H660‐H664, H670, H671, H70, H750, H940, J32, J340, J36, J378, J390, J391, K040, K041, K044, K046, K047, K052, K113, K112 | Ear, nose and throat infections |
| I301, I320, I321, I33, I38, I400, I410‐I412, I980, I981 | Cardiovascular system infections |
| J00x-J06x | Acute upper respiratory infections |
| J09x-J18x, J20-J22, J850-J852, J86 | Pulmonary infections |
| D733, K230, K231, K35‐K37, K57, K61, K630, K631, K65, K67, K750, K770, K800, K801, K803, K81, K871, K930, K931 | Gastro‐intestinal infections |
| L00‐L08 | Dermatological infections |
| M00, M01, M600, M630‐M632, M650, M651, M680, M710, M711, M730, M731, M86, M900‐M902 | Musculoskeletal system infections |
| N080, N10, N12, N136, N151, N160, N220, N290, N291, N300, N33x, N34x, N390, N41x, N431x, N45x, N481, N482, N49x, N51x, N61x, N70x‐N76x N770, N771 | Urinary and gynecologic tract infections |
| O23, O753, O85, O86, O91, O98 | Maternal infections during pregnancy |
| U80, U81, U88, U89 | Others |
| **Thrombotic events** | |
| I26x | Pulmonary embolism |
| I80x | Phlebitis and thrombophlebitis |
| I81 | Portal vein thrombosis |
| I82x | Other venous embolism and thrombosis |
| I676 | Nonpyogenic thrombosis of intracranial venous system |
| I63x | Cerebral infarction |
| I21x | Acute myocardial infarction |
| I240 | Coronary thrombosis not resulting in myocardial infarction |
| I65x | Occlusion and stenosis of precerebral arteries, not resulting in cerebral infarction |
| I66x | Occlusion and stenosis of cerebral arteries, not resulting in cerebral infarction |
| G45x | Transient cerebral ischemic attacks and related syndromes |
| I74x | Arterial embolism and thrombosis |
| I75x | Atheroembolism |
| K550 | Acute vascular disorders of intestine |
| K551 | Chronic vascular disorders of intestine |
| K559 | Vascular disorders of intestine, unspecified |
| G951 | Vascular myelopathies |
| H34x | Retinal vascular occlusions |
| K763 | Infarction of liver |
| N280 | Ischaemia and infarction of kidney |
| **Skeletal related events** | |
| M80-M94 | Osteopathies and chondropathies |
| **Neuropathy** | |
| G620 | Drug-induced polyneuropathy |
| **Non-infectious or non-specified diarrhoea** | |
| K529 | Noninfective gastroenteritis and colitis, unspecified |
| K591 | Functional diarrhoea |
| A099 | Gastroenteritis and colitis of unspecified origin |
| **Shingles** | |
| B02 | Zoster (incuding shingles, zona) |
| **Pneumonia** | |
| J12.0 - J18 | Pneumonia |

AE, adverse events; ICD-10, the International Classification of Diseases version 10; NOS, not otherwise specified.

## Supplementary Table 4. Distribution of study patients by index date and diagnosis type

| **Year of index date** | **Patients with two hospitalization records** | | **Patients with one long-term disease record and one hospitalization record** | | **Total patients enrolled** | |
| --- | --- | --- | --- | --- | --- | --- |
|  | **n** | **%** | **n** | **%** | **n** | **%** |
| 2013 | 508 | 52.0 | 469 | 48.0 | 977 | 15.2 |
| 2014 | 549 | 53.8 | 471 | 46.2 | 1020 | 15.9 |
| 2015 | 564 | 54.5 | 471 | 45.5 | 1035 | 16.1 |
| 2016 | 605 | 54.6 | 504 | 45.4 | 1109 | 17.3 |
| 2017 | 629 | 51.3 | 597 | 48.7 | 1226 | 19.1 |
| 2018 | 495 | 47.3 | 551 | 52.7 | 1046 | 16.3 |
| All years | 3350 | 52.2 | 3063 | 47.8 | 6413 | 100.0 |

**Supplementary Table 5. Patient comorbidities**

| **Comorbidity** | **Patients with SCT (n=1910)** | | **Patients  without SCT (n=4503)** | | **Total patients  (N=6413)** | |
| --- | --- | --- | --- | --- | --- | --- |
|  | **n** | **%** | **n** | **%** | **n** | **%** |
| Cancer  (including lymphoma and leukaemia) | 1910 | 100 | 4503 | 100 | 6413 | 100 |
| Diabetes  With target organ involvement | 164  27 | 8.6  1.4 | 901  229 | 20.0  5.1 | 1065  256 | 16.6  4.0 |
| Moderate or severe renal diseases | 137 | 7.2 | 898 | 19.9 | 1035 | 16.1 |
| Chronic pulmonary disease | 144 | 7.5 | 665 | 14.8 | 809 | 12.6 |
| Congestive heart failure | 26 | 1.4 | 505 | 11.2 | 531 | 8.3 |
| Myocardial infarction | 19 | 1.0 | 216 | 4.8 | 235 | 3.7 |
| Obliterating arteriopathy of lower limbs | 20 | 1.0 | 161 | 3.6 | 181 | 2.8 |
| Cerebrovascular accident | 9 | 0.5 | 155 | 3.4 | 164 | 2.6 |
| Mild hepatic disease | 38 | 2.0 | 105 | 2.3 | 143 | 2.2 |
| Dementia | 1 | 0.1 | 125 | 2.8 | 126 | 2.0 |
| Hemiplegia | 16 | 0.8 | 93 | 2.1 | 109 | 1.7 |
| Gastro-duodenal peptic ulcer disease | 10 | 0.5 | 55 | 1.2 | 65 | 1.0 |
| Connectivity | 4 | 0.2 | 39 | 0.9 | 43 | 0.7 |
| Moderate or severe hepatic disease | 2 | 0.1 | 30 | 0.7 | 32 | 0.5 |
| AIDS | 5 | 0.3 | 8 | 0.2 | 13 | 0.2 |

AIDS, acquired immunodeficiency syndrome; SCT, stem cell transplant.

**Supplementary Table 6. Numbers of study patients and deaths during follow-up by SCT status**

| **LOT at end of follow-up** | **Patients with SCT** | | | | **Patients without SCT** | | | |  |
| --- | --- | --- | --- | --- | --- | --- | --- | --- | --- |
|  | **Total patients**  **(n=1910)** | | **Deceased patients** | **% LOT deceased** | **Total patients**  **(n=4503)** | | **Deceased patients** | **% LOT deceased** |  |
|  | **n** | **%** | **n** | **%** | **n** | **%** | n | % |  |
| Undetermined | 27 | 1.4 | 4 | 14.8 | 157 | 3.5 | 71 | 45.2 |  |
| 1 | 663 | 34.7 | 33 | 5.0 | 1891 | 42.0 | 804 | 42.5 |  |
| 2 | 565 | 29.6 | 58 | 10.3 | 1214 | 27.0 | 429 | 35.3 |  |
| 3 | 306 | 16.0 | 53 | 17.3 | 604 | 13.4 | 254 | 42.1 |  |
| 4 | 129 | 6.8 | 28 | 21.7 | 278 | 6.2 | 135 | 48.6 |  |
| 5+ | 220 | 11.5 | 103 | 89.3 | 359 | 8.0 | 195 | 105.9 |  |

LOT, line of treatment; SCT, stem cell transplant.

## Supplementary Table 7. Treatment regimens used across LOTs

| **Category** | **Included regimens** | **LOT1 (n=6229)** | | **LOT2 (n=3675)** | | **LOT3 (n=1896)** | | **LOT4 (n=986)** | | **LOT5+ (n=1469)** | |
| --- | --- | --- | --- | --- | --- | --- | --- | --- | --- | --- | --- |
|  |  | n | % | n | % | n | % | n | % | n | % |
| Bortezomib | Bortezomib/cyclophosphamide | 3107 | 25 | 355 | 5 | 156 | 4 | 91 | 5 | 118 | 4 |
|  | Bortezomib |  |  |  |  |  |  |  |  |  |  |
|  | Bortezomib/doxorubicin |  |  |  |  |  |  |  |  |  |  |
| Bortezomib/melphalan | Bortezomib/melphalan | 1055 | 8 | 820 | 11 | 38 | 1 | 18 | 1 | 15 | 1 |
| Bortezomib/thalidomide | Bortezomib/thalidomide | 1408 | 11 | 264 | 4 | 46 | 1 | 6 | 0 | 8 | 0 |
| Bortezomib/lenalidomide | Bortezomib/thalidomide/lenalidomide | 456 | 4 | 586 | 8 | 222 | 6 | 68 | 3 | 28 | 1 |
|  | Bortezomib/lenalidomide |  |  |  |  |  |  |  |  |  |  |
|  | Bortezomib/melphalan/lenalidomide |  |  |  |  |  |  |  |  |  |  |
| Lenalidomide | Lenalidomide/cyclophosphamide | 66 | 1 | 1213 | 17 | 583 | 15 | 113 | 6 | 104 | 4 |
|  | Lenalidomide |  |  |  |  |  |  |  |  |  |  |
|  | Melphalan/lenalidomide |  |  |  |  |  |  |  |  |  |  |
| Pomalidomide | Lenalidomide/pomalidomide | 2 | 0 | 51 | 1 | 243 | 6 | 232 | 12 | 279 | 9 |
|  | Pomalidomide |  |  |  |  |  |  |  |  |  |  |
|  | Pomalidomide/bortezomib |  |  |  |  |  |  |  |  |  |  |
|  | Pomalidomide/cyclophosphamide |  |  |  |  |  |  |  |  |  |  |
| Daratumumab | Daratumumab/pomalidomide | 7 | 0 | 47 | 1 | 123 | 3 | 151 | 8 | 319 | 11 |
|  | Daratumumab |  |  |  |  |  |  |  |  |  |  |
|  | Daratumumab/bortezomib/melphalan |  |  |  |  |  |  |  |  |  |  |
|  | Daratumumab/bortezomib |  |  |  |  |  |  |  |  |  |  |
|  | Daratumumab/lenalidomide |  |  |  |  |  |  |  |  |  |  |
| Carfilzomib | Daratumumab/carfilzomib | 1 | 0 | 33 | 0 | 58 | 2 | 30 | 2 | 91 | 3 |
|  | Carfilzomib/cyclophosphamide |  |  |  |  |  |  |  |  |  |  |
|  | Carfilzomib |  |  |  |  |  |  |  |  |  |  |
|  | Pomalidomide/carfilzomib |  |  |  |  |  |  |  |  |  |  |
|  | Carfilzomib/lenalidomide |  |  |  |  |  |  |  |  |  |  |
| Ixazomib | Ixazomib | 0 | 0 | 18 | 0 | 62 | 2 | 24 | 1 | 23 | 1 |
|  | Ixazomib/cyclophosphamide |  |  |  |  |  |  |  |  |  |  |
|  | Ixazomib/lenalidomide |  |  |  |  |  |  |  |  |  |  |
|  | Ixazomib/pomalidomide |  |  |  |  |  |  |  |  |  |  |
| Bendamustine | Bendamustine | 13 | 0 | 25 | 0 | 60 | 2 | 59 | 3 | 89 | 3 |
|  | Bendamustine/bortezomib |  |  |  |  |  |  |  |  |  |  |
|  | Bendamustine/lenalidomide |  |  |  |  |  |  |  |  |  |  |
| Other | Melphalan/thalidomide | 114 | 1 | 263 | 4 | 305 | 8 | 194 | 10 | 395 | 13 |
|  | Thalidomide |  |  |  |  |  |  |  |  |  |  |
|  | Melphalan |  |  |  |  |  |  |  |  |  |  |
|  | Cyclophosphamide |  |  |  |  |  |  |  |  |  |  |
|  | Doxorubicin |  |  |  |  |  |  |  |  |  |  |
|  | Other |  |  |  |  |  |  |  |  |  |  |

Dexamethasone and prednisone were not always observed (owing to incomplete data availability), so for some patients use in combination with other treatments is assumed.

LOT, line of treatment.

## Supplementary Table 8. Median treatment duration (in months*) by LOT and SCT status

|  | **SCT - LOT1** | | | **No SCT - LOT1** | | |
| --- | --- | --- | --- | --- | --- | --- |
| **Regimen**^†^ | **n** | **%** | **Median (IQR), months** | **n** | **%** | **Median (IQR)** |
| **Bortezomib-based^‡^** | 1624 | 86% | 9.3 (5.2, 17.1) | 3946 | 91% | 5.3 (2.2, 12.5) |
| **Bortezomib + Lenalidomide-based** | 222 | 12% | 11.3 (5.2, 25.4) | 234 | 5% | 7.8 (4.5, 17.7) |
| **Other** | 37 | 2% | 2.4 (2.2, 6) | 166 | 4% | 7.4 (2.4, 20.5) |
| **LOT1 total** | 1883 | 100% | 9.3 (5.1, 17.9) | 4346 | 100% | 5.6 (2.3, 12.8) |
|  | **SCT - LOT2** | | | **No SCT - LOT2** | | |
| **Regimen** | **n** | **%** | **Median (IQR)** | **n** | **%** | **Median (IQR)** |
| **Lenalidomide-based** | 447 | 37% | 12.1 (4.8, 21.7) | 1085 | 44% | 10.0 (4.6, 16.8) |
| **Bortezomib + Lenalidomide-based** | 253 | 21% | 8.4 (4.0, 18.4) | 766 | 31% | 11.4 (5.0, 23.8) |
| **Bortezomib-based** | 354 | 29% | 5.3 (3.1, 15.5) | 333 | 14% | 7.4 (3.1, 15.6) |
| **Other** | 166 | 14% | 2.3 (1.2, 8.8) | 271 | 11% | 2.7 (1.3, 8.4) |
| **LOT2 total** | 1220 | 100% | 7.7 (3.3, 19) | 2455 | 100% | 9.4 (3.7, 17.6) |
|  | **SCT - LOT3** | | | **No SCT - LOT3** | | |
| **Regimen** | **n** | **%** | **Median (IQR)** | **n** | **%** | **Median (IQR)** |
| **Lenalidomide-based** | 134 | 20% | 12.6 (4.6, 23.7) | 449 | 36% | 10.8 (4.2, 21.2) |
| **Pomalidomide-based** | 87 | 13% | 4.3 (2.4, 14.0) | 156 | 13% | 5.1 (2.1, 11.1) |
| **Bortezomib + Lenalidomide-based** | 104 | 16% | 13.2 (5.3, 22.1) | 118 | 10% | 3.9 (1.6, 12.5) |
| **Other** | 330 | 50% | 2.8 (1.2, 8.8) | 518 | 42% | 2.9 (1.2, 8.1) |
| **LOT3 total** | 655 | 100% | 5.4 (1.7, 15.6) | 1241 | 100% | 5.3 (1.7, 14.3) |
|  | **SCT - LOT4** | | | **No SCT – LOT 4** | | |
| **Regimen** | **n** | **%** | **Median (IQR)** | **n** | **%** | **Median (IQR)** |
| **Daratumumab-based** | 52 | 15% | 4.3 (1.9, 9.8) | 99 | 16% | 4.9 (1.5, 11.6) |
| **Pomalidomide-based** | 86 | 25% | 2.8 (1.6, 7.4) | 146 | 23% | 3.1 (1.5, 6.8) |
| **Lenalidomide-based** | 41 | 12% | 15.6 (4.3, 29.7) | 72 | 11% | 7.6 (2.8, 17.8) |
| **Bortezomib-based** | 39 | 11% | 4.7 (1.2, 9.1) | 76 | 12% | 3.7 (1.4, 8.6) |
| **Other** | 131 | 38% | 2.0 (1.1, 7.1) | 244 | 38% | 1.8 (1.0, 5.4) |
| **LOT4 total** | 349 |  | 3.5 (1.3, 10.4) | 637 |  | 3 (1.2, 8.4) |
|  | **SCT - LOT5+** | | | **No SCT – LOT 5+** | | |
| **Regimen** | **n** | **%** | **Median (IQR)** | **n** | **%** | **Median (IQR)** |
| **Daratumumab-based** | 147 | 42% | 2.5 (1.3, 6.0) | 172 | 27% | 3.0 (1.2, 6.6) |
| **Pomalidomide-based** | 113 | 32% | 3.7 (1.6, 6.9) | 166 | 26% | 2.6 (1.4, 6.8) |
| **Lenalidomide-based** | 43 | 12% | 3.6 (2.1, 10.7) | 61 | 10% | 4.7 (1.5, 10.5) |
| **Bortezomib-based** | 50 | 14% | 2.6 (1.1, 4.6) | 91 | 14% | 3.2 (1.2, 7.6) |
| **Other** | 274 | 79% | 1.4 (0.9, 2.2) | 352 | 55% | 1.6 (1.0, 3.2) |
| **LOT5+ total** | 627 |  | 2.0 (1.0, 4.7) | 842 |  | 2.3 (1.1, 5.3) |

*Median duration and interquartile range were calculated using Kaplan-Meier method, with patients censored when lost to follow-up or reaching the end of the study period.

†A limitation of the algorithm that excluded partially observable drugs, such as melphalan and cyclophosphamide, may have resulted in erroneous underestimation of LOT1 duration for all patients, and LOT2 duration in patients without SCT who received a bortezomib-based regimen.

^‡^For all LOTs, bortezomib-based regimens included bortezomib (alone or in combination with cyclophosphamide or doxorubicin), bortezomib/melphalan, and bortezomib/thalidomide.

IQR, interquartile range; LOT, line of treatment; SCT, stem cell transplant.

## Supplementary Table 9. HCRU per person per month by LOT

| **HCRU** | **LOT1** | | | **LOT2** | | | **LOT3** | | | **LOT4** | | | **LOT5+** | | | |
| --- | --- | --- | --- | --- | --- | --- | --- | --- | --- | --- | --- | --- | --- | --- | --- | --- |
|  | **(n=6229)** | | | **(n=3675)** | | | **(n=1896)** | | | **(n=986)** | | | **(n=579)** | | | |
|  | **n** | **%** | **PPPM, days** | **n** | **%** | **PPPM, days** | **n** | **%** | **PPPM, days** | **n** | **%** | **PPPM, days** | **n** | **%** | **PPPM, days** |  |
| Total MCO hospitalisations | 4692 | 75.3 | 2.3 | 2327 | 63.3 | 1.7 | 1169 | 61.7 | 1.8 | 582 | 59.0 | 2.3 | 476 | 82.2 | 3.0 |  |
| Complete MCO hospitalisations | 4002 | 64.2 | 2.2 | 1834 | 49.9 | 1.6 | 906 | 47.8 | 1.8 | 429 | 43.5 | 2.2 | 411 | 71.0 | 3.0 |  |
| Chemotherapy sessions - ambulatory (PPPM on number of sessions) | 5742 | 92.2 | 1.6 | 2374 | 64.6 | 0.9 | 903 | 47.6 | 0.7 | 523 | 53.0 | 0.9 | 415 | 71.7 | 1.2 |  |
| Chemotherapy sessions - hospitalisation for chemotherapy (> 1 day) | 1369 | 22.0 | 0.4 | 375 | 10.2 | 0.2 | 199 | 10.5 | 0.2 | 144 | 14.6 | 0.3 | 176 | 30.4 | 0.5 |  |
| Hospitalisation at home | 857 | 13.8 | 0.8 | 325 | 8.8 | 0.3 | 106 | 5.6 | 0.3 | 64 | 6.5 | 0.4 | 67 | 11.6 | 0.3 |  |
| Hospitalisation in rehabilitation centre | 1033 | 16.6 | 1.1 | 283 | 7.7 | 0.4 | 144 | 7.6 | 0.5 | 61 | 6.2 | 0.5 | 60 | 10.4 | 0.5 |  |
| Emergency stays | 1071 | 17.2 | 0.0 | 632 | 17.2 | 0.0 | 259 | 13.7 | 0.0 | 104 | 10.5 | 0.0 | 102 | 17.6 | 0.0 |  |

HCRU, health care resource utilisation; LOT, line of treatment; MCO, medical, surgical, and obstetric; MM, multiple myeloma; PPPM, per person per month.

## Supplementary Table 10. Cost of hospitalisations related to events of interest (AEs and comorbidity) associated with primary diagnosis of MM annualised to 2019

| **Population** | **Total cost of hospitalisations of patients** | | | | |
| --- | --- | --- | --- | --- | --- |
|  | **n** | **Cost, M**€ | **%** | **Cost rate PP, K^a^** | **Cost PPPM, K** |
| Total population events (n=6413)  During first year | 7397  4463 | 29.6  17.9 | 9.5  7.9 | 2.1  – | –  0.28 |
| SCT events (n=1910)  During first year | 2090  1233 | 9.9  6.1 | 8.0  6.4 | 1.9  – | –  0.28 |
| LOT1 events (n=6229) | 3203 | 12.2 | 8.5 | 0.2 | – |
| LOT2 events (n=3675) | 1478 | 5.5 | 11.1 | 0.1 | – |
| LOT3 events (n=1896) | 654 | 2.9 | 14.5 | 0.2 | – |
| LOT4 events (n=986) | 331 | 1.4 | 14.8 | 0.3 | – |
| LOT5+ events (n=579) | 443 | 1.9 | 14.6 | 0.3 | – |

^a^Rate was per PPPY for total population and SCT subgroup and PPPM for LOT subgroups.
AE, adverse events; K, thousands; LOT, line of treatment; M, millions; MM, multiple myeloma; PP, per patient; PPPM, per patient per month; PPPY, per patient per year; SCT, stem cell transplantation.

## Supplementary Table 11. Cost of HCRU related only to MM

|  | **LOT1**  **(n=6229)** | | **LOT2**  **(n=3675)** | | **LOT3**  **(n=1896)** | | **LOT4**  **(n=986)** | | **LOT5+**  **(n=579)** | | **Patients with SCT**  **(n=1910)** | | **Total**  **(N=6413)** | |
| --- | --- | --- | --- | --- | --- | --- | --- | --- | --- | --- | --- | --- | --- | --- |
|  | **Sum, M€** | **Monthly rate, K€** | **Sum, M€** | **Monthly rate, K€** | **Sum, M€** | **Monthly rate, K€** | **Sum, M€** | **Monthly rate, K€** | **Sum, M€** | **Monthly rate, K€** | **Overall** | **First year**  **PPPY** | **Overall** | **First year**  **PPPY** |
| Total MM MCO hospitalisation | 68.68 | 1.136 | 22.40 | 0.577 | 11.13 | 0.778 | 5.40 | 1.011 | 7.52 | 1.313 | 83.52 | 66.90 | 168.15 | 127.56 |
| Hospitalisation at home | 8.56 | 0.142 | 2.36 | 0.061 | 0.70 | 0.049 | 0.34 | 0.063 | 0.33 | 0.058 | 3.63 | 2.94 | 13.40 | 10.40 |
| Hospitalisation in rehabilitation centre | 10.20 | 0.169 | 1.95 | 0.050 | 0.74 | 0.052 | 0.36 | 0.067 | 0.27 | 0.047 | 2.91 | 2.43 | 15.96 | 12.86 |
| Transport (following MM hospitalisation) | 0.39 | 0.006 | 0.08 | 0.002 | 0.04 | 0.002 | 0.02 | 0.004 | 0.03 | 0.006 | 0.42 | 0.37 | 0.83 | 0.71 |
| Sick leave (7 days following MM hospitalisation) | 0.77 | 0.013 | 0.11 | 0.003 | 0.04 | 0.003 | 0.02 | 0.004 | 0.02 | 0.004 | 1.05 | 0.93 | 1.16 | 1.04 |
| Total cost of treatment related to MM | 147.96 | 2.447 | 110.80 | 2.852 | 62.45 | 4.369 | 32.10 | 6.014 | 40.26 | 7.026 | 154.84 | 73.11 | 412.01 | 214.55 |
| **Total cost of MM patient** | **236.56** | **3.913** | **137.70** | **3.544** | **75.08** | **5.254** | **38.23** | **7.162** | **48.43** | **8.452** | **246.38** | **146.69** | **611.50** | **367.11** |

HCRU, health care resource utilisation; K, thousands; LOT, line of treatment; M, millions; MCO, medical, surgical, and obstetric; MM, multiple myeloma; SCT, stem cell transplant.
